# Software-Defined-Networking-Based One-versus-Rest Strategy for Detecting and Mitigating Distributed Denial-of-Service Attacks in Smart Home Internet of Things Devices

**DOI:** 10.3390/s24155022

**Published:** 2024-08-03

**Authors:** Neder Karmous, Mohamed Ould-Elhassen Aoueileyine, Manel Abdelkader, Lamia Romdhani, Neji Youssef

**Affiliations:** 1 Innov’COM Laboratory, Higher School of Communication of Tunis (SUPCOM), Technopark Elghazala, Raoued, Ariana 2083, Tunisia; nader.karmous@supcom.tn (N.K.); mohamed.ouldelhassen@supcom.tn (M.O.-E.A.); neji.youssef@supcom.tn (N.Y.); 2 Computer Science Department, Tunis Business School, University Tunis Elmanar, Tunis 1068, Tunisia; manel.abdelkader@gmail.com; 3 Core Curriculum Program, Deanship of General Studies, University of Qatar, Doha P.O. Box 2713, Qatar

**Keywords:** internet of things, intrusion detection system, smart home, software-defined networking, machine learning, artificial intelligence, deep learning, cyber security, distributed denial of service

## Abstract

The number of connected devices or Internet of Things (IoT) devices has rapidly increased. According to the latest available statistics, in 2023, there were approximately 17.2 billion connected IoT devices; this is expected to reach 25.4 billion IoT devices by 2030 and grow year over year for the foreseeable future. IoT devices share, collect, and exchange data via the internet, wireless networks, or other networks with one another. IoT interconnection technology improves and facilitates people’s lives but, at the same time, poses a real threat to their security. Denial-of-Service (DoS) and Distributed Denial-of-Service (DDoS) attacks are considered the most common and threatening attacks that strike IoT devices’ security. These are considered to be an increasing trend, and it will be a major challenge to reduce risk, especially in the future. In this context, this paper presents an improved framework (SDN-ML-IoT) that works as an Intrusion and Prevention Detection System (IDPS) that could help to detect DDoS attacks with more efficiency and mitigate them in real time. This SDN-ML-IoT uses a Machine Learning (ML) method in a Software-Defined Networking (SDN) environment in order to protect smart home IoT devices from DDoS attacks. We employed an ML method based on Random Forest (RF), Logistic Regression (LR), k-Nearest Neighbors (kNN), and Naive Bayes (NB) with a One-versus-Rest (OvR) strategy and then compared our work to other related works. Based on the performance metrics, such as confusion matrix, training time, prediction time, accuracy, and Area Under the Receiver Operating Characteristic curve (AUC-ROC), it was established that SDN-ML-IoT, when applied to RF, outperforms other ML algorithms, as well as similar approaches related to our work. It had an impressive accuracy of 99.99%, and it could mitigate DDoS attacks in less than 3 s. We conducted a comparative analysis of various models and algorithms used in the related works. The results indicated that our proposed approach outperforms others, showcasing its effectiveness in both detecting and mitigating DDoS attacks within SDNs. Based on these promising results, we have opted to deploy SDN-ML-IoT within the SDN. This implementation ensures the safeguarding of IoT devices in smart homes against DDoS attacks within the network traffic.

## 1. Introduction

Traditional network architectures [1], where switches and routers combine control and data planes, rely on distributed control and static configurations, often require manual configurations, and lack the agility to swiftly adapt to evolving IoT network demands. SDN [2] is a network concept that revolutionizes traditional network architectures for managing and securing computer networks, smart grids, data centers, and, especially, IoT devices by decoupling the control plane from the data plane. In an SDN for IoT, a centralized controller orchestrates IoT network devices, allowing dynamic configuration and adaptability through software-defined policies. It also offers unparalleled flexibility and scalability. This separation of control enables rapid innovation, vendor independence, and efficient resource utilization tailored for IoT environments; automation is intrinsic to SDNs for IoT, streamlining tasks such as provisioning and optimization. In this context, we used an SDN as an approach to make IoT networks more adaptable and flexible by improving network control, management, and security. We combined an SDN with ML [3,4,5] to enhance the security of IoT devices in a smart home. We used an SDN control plane, SDN data plane, and ML model as an approach to building IoT network security. The SDN control plane centralizes network intelligence and management, making it more flexible and programmable. The SDN data plane, often implemented in network switches and routers, is responsible for forwarding data packets based on instructions received from the SDN controller in the control plane. It follows the policies and rules set by the controller. The ML model, after training data, can predict potential threats and vulnerabilities in the IoT network. It can also adapt and learn from new data to improve prediction accuracy. When a security threat is detected, the SDN controller, in collaboration with the ML model, can trigger automated responses. This helps mitigate the impact on the affected IoT device and alerts network administrators. The OpenFlow (OF) protocol [6] enables the OF Controller to instruct the OF switch on how to handle incoming data packets; it adds flow entries from the switch’s flow tables [7], specifying the criteria to perform an action, which is, in our case, dropping the packet. Upon receiving the flow modification message, the OF switch installs the flow entry into its flow table, and it will start blocking traffic that matches the specified criteria. The objective of this paper is to achieve the real-time detection and mitigation of DDoS attacks [8,9] originating from smart home IoT devices within a Software-Defined Networking (SDN) environment. This is accomplished through the implementation of the SDN-ML-IoT method, which is based on supervised ML and is capable of detecting multiple DDoS attacks that pose a genuine threat to IoT devices. SDN-ML-IoT utilizes diverse approaches to ensure the accuracy and suitability of data, resulting in enhanced convergence and model optimization. These approaches include Recursive Feature Elimination (RFE), cross-validation k-fold, and undersampling for balancing data. One of the major challenges in this process is distinguishing between malicious and legitimate traffic. To address this, we employ the OvR strategy, which simplifies the multiclass classification problem by breaking it down into a series of binary classification tasks. This approach facilitates the distinction between different classes, streamlining the overall classification process. Let us summarize our paper, which consists of five main sections. In the first section, we present an overview of the work related to our SDN-ML-IoT method. The second section delves into the background, which serves to provide readers with a comprehensive understanding of the context motivations and existing knowledge related to our research topic. The third section outlines the methodology employed to build the SDN-ML-IoT framework, and we present an analysis of the results obtained based on ML algorithms specializing in IDPS [10] and the security of IoT devices [11] utilizing evaluation metrics. These include RF [12], LR [13], KNN [14] and NB [15]. In the fourth section, we deploy our SDN framework in a live network and subject it to comprehensive testing to evaluate its performance, effectiveness, and reliability in detecting and mitigating DDoS attacks in real-world scenarios. Finally, we compare the results of SDN-ML-IoT with those achieved in the related works.

## 2. Related Works

The study in  [16] proposed a DDoS attack detection method that uses conditional entropy [17] based on SDN traffic to reduce the incidence of false positives rate. The author uses Scapy [18] to generate normal and DDoS traffic, flash Crowds, ICMP flooding and packet-in attacks. The proposed method for identifying anomalous DDoS traffic quantifies the concentration of traffic based on the mean and standard deviation and uses changes in three types of entropy values to determine the type of traffic, thereby achieving more precise attack detection. Additionally, pre-processing is performed during traffic collection, so it is not necessary to traverse all collected packets but only to process a random sample of packets to quickly obtain entropy values while maintaining a certain level of accuracy. This approach has lower false positive rates, a higher detection accuracy at 97.2%, and faster response times at 0.74 s. The limitations of this work include the need to enhance accuracy, develop effective mitigation methods for countering DDoS attacks post-detection, and validate the deployment of these methods in real traffic to assess their efficiency.

The author in [19] introduces a novel Secured Automatic Two-level Intrusion Detection System, called SATIDS, which leverages an enhanced Long–Short-Term Memory (LSTM) network [20]. SATIDS’s primary objective is to effectively distinguish between malicious attacks and benign network traffic, accurately identify attack categories, and specify sub-attack types with exceptional performance. The approach proposed in this paper is assessed using the ToN-IoT dataset [21], encompassing network traffic data from various IoT devices and scenarios that simulate real-world IoT network traffic. Additionally, the InSDN dataset [22] is utilized, which includes several types of DoS attacks across different OSI model layers. To execute various DoS attacks, Kali Linux is employed against a victim web server represented by an h4 virtual host, including TCP, UDP, and HTTP flood attacks, through the Low Orbit Ion Cannon (LOIC) tool [23]. The experimental results reveal that when facing DDOS attacks using the ToN-IoT dataset, the SATIDS system performs optimally with 3 LSTM layers and 500 hidden layers, achieving 94.8% precision and a 92.7% detection rate. For the INSDN DATASET, utilizing 3 LSTM layers and 500 hidden layers yields a precision rate of 90% for DDOS attacks. This work has limitations, including the need for further accuracy improvements and the fact that it can only detect attacks without mitigation. Further testing and deployment of the SATIDS model in real traffic networks are necessary.

Singh, C  [24] proposed a method for detecting DDoS attacks in SDN using the Gini impurity [25]. The approach is specifically designed for IoT networks, taking advantage of centralized control and efficient security threat management. To create a CSV dataset for normal and DDoS attacks, the author used the CICFlowMeter program [26] to create it and selected 42 features out of 80 based on their correlation matrix score. The proposed method was evaluated on the NSL-KDD dataset [27], which contains three types of DDoS attacks: UDP, ICMP, and TCP attacks. They applied four ML algorithms—Multilayer Perceptron (MLP) [28], LR, kNN and Decision Tree (DT) [29]—along with their proposed Gini-impurity-based approach to test the performance of these algorithms. The Gini impurity method achieved an impressive accuracy of 99.9%. Moreover, the proposed approach not only detects DDoS attacks but also includes effective mitigation strategies. Finally, the method was successfully deployed on an SDN network, further validating its practical applicability. The work looks promising, but it could benefit from employing feature reduction algorithms to further reduce the number of features and considering the use of a multiclass approach to detect different types of DDoS attacks.

The researcher in [30] introduced a DDoS detection method leveraging feature engineering and ML within SDN. The CSE-CIC-IDS2018 dataset [31] was employed, and 26 significant features were selected from an initial set of 79 using the binary grey wolf optimization algorithm [32]. Consequently, SVM [33], RF, Decision Tree, XGBoost [34], and kNN were utilized to assess and determine the best classifier for both the original and feature-extracted datasets. All classifiers demonstrated improvement across various metrics. Notably, the RF classifier outperformed others in terms of accuracy (0.9913), precision (0.9843), recall (0.9992), and f1-score (0.9913). Following the deployment of the best classifier selection method based on the RF model to the controller, DDoS detection was executed using features from a subset of the most influential features. The results affirmed the capability of the proposed method to detect DDoS attacks and alert users in real time. However, the study acknowledges a limitation, indicating the need for enhancing classifier performance and accuracy, reducing features, and testing the network on various SDN topologies to assess its efficiency.

The research paper in [35] proposed a method for detecting DDoS attacks in SDN security. This work introduces RF, kNN, NB, and LR as supervised ML algorithms for DDoS attack detection across three distinct network architectures: single topology, linear topology, and multi-controller topology. The models are trained using datasets generated in a simulated SDN environment utilizing the Mininet emulator and Ryu controller. The simulation results reveal that NB and LR exhibit low accuracy rates, generating numerous incorrect predictions. In contrast, RF and KNN demonstrate high accuracy rates and are deemed effective prediction models for this study. Observations made during the attack, based on monitoring network traffic, indicate that the assault primarily aims to exhaust the controller and induce its failure by inundating the switch flow table with requests containing spoofed IP addresses. Additionally, it leads to some disruption in normal packet flow as the controller is occupied with these falsified requests. This impact is predominantly noticeable in the single topology, as opposed to linear and multi-controller topologies, suggesting that increasing the number of switches reduces the load and facilitates rapid elimination of the attack effect. Moreover, augmenting network switches minimizes detection and mitigation times. Furthermore, an increase in the number of controllers enhances the detection and mitigation process by reducing error rates, detection times, and mitigation times. Ultimately, the proposed mitigation technique is successfully implemented to thwart the attack before causing harm to the controller by blocking the attacker port for 120 s. However, it seems like this work exhibits overfitting in accuracy results and requires the implementation of feature selection methods along with cross-validation and other approaches to mitigate overfitting in accuracy outcomes.

Karthika, P  [36] proposed architecture based on OF port statistics for implementing ML-enhanced TCP/SYN flood detection and mitigation. The author employed ML techniques, including SVM, NB, and MLP. A total of 6 features were carefully selected from 25 to effectively distinguish between regular traffic and SYN flood traffic. Additionally, the method mitigates the impacts of the attacking node on the network by utilizing the MAC address of the host. The results indicate that the MLP achieved the highest classification accuracy, reaching 99.75% for the simulation dataset. However, this work needs to focus on other protocols capable of targeting and collecting a broader range of normal and DDoS data. These protocols should have the potential to impact various ports, such as HTTP/HTTPS, Message Queuing Telemetry Transport (MQTT), and Constrained Application Protocol (CoAP).

The author in [37] presented FMDADM, an ML-based DDoS detection and mitigation framework tailored for SDN-enabled IoT networks. The framework comprises three detection modules and a mitigation module. Notably, it employs a 32-packet window size, a novel mapping function (DCMF), and feature engineering to enhance accuracy and address overfitting. The proposed framework, evaluated with various ML models, demonstrated superior performance, particularly with the RF model. FMDADM effectively detects DDoS attacks in multi-node scenarios, showcasing strength where conventional defenses may fall short. The framework is designed to prevent local IoT Botnet-induced DDoS attacks from reaching the ISP level, offering protection to the controller and remote nodes. The experimental results show that FMDADM surpasses current solutions in terms of accuracy, precision, F-measure, recall, specificity, negative predictive value, false positive rate, false detection rate, false negative rate, and average detection time, achieving 99.79%, 99.43%, 99.77%, 99.79%, 99.95%, 0.21%, 0.91%, 0.23%, and 2.64 μs, respectively.

The authors of  [38] demonstrate the effectiveness of employing deep learning methods, specifically a hybrid model combining 1D Convolutional Neural Network (CNN), Gated Recurrent Unit (GRU), and Dense Neural Network (DNN), to detect and protect against DDoS attacks in SDN environments. The proposed model outperforms traditional ML algorithms in accurately identifying DDoS attacks, especially low-rate ones, and detecting both short-term and long-term patterns in input data. However, limitations include the evaluation of a specific dataset, necessitating further testing on diverse datasets and network topologies for generalizability. Future research should focus on effective mitigation strategies post-detection. Despite these considerations, the findings underscore the importance of employing deep learning techniques for DDoS detection and defense in SDN networks. The hybrid model is identified as a valuable tool contributing to overall security and stability, with future research recommended to explore additional strategies for further enhancing detection and response to DDoS attacks in SDN networks.

The summary of related work in relation to our research is presented in Table 1 below.

## 3. Background

In this section, our focus is on elucidating the orientation of our paper, centered on the objective related to smart home technology [39,40]. Firstly, we define the MQTT protocol, which is utilized in the majority of IoT devices for smart homes [41]. MQTT stands out in smart home applications due to its speed, reliability, security, and compatibility with a broad array of devices and platforms [42]. Secondly, we delve into the various types of DDoS attacks that pose threats to these IoT devices, presenting a diverse set of algorithms that showcase effectiveness in detecting DDoS attacks [43,44,45]. Lastly, we employed the integration of SDN into the IoT network infrastructure, utilizing an ML-based Ryu SDN Controller to improve the detection and mitigation of DDoS attacks. This strategy provides a proactive defense mechanism against DDoS attacks directed at IoT devices, thereby enhancing the security and resilience of our smart home environment.

### 3.1. IoT for Smart Homes

#### 3.1.1. MQTT Protocol Overview

- MQTT: This is a lightweight messaging protocol designed for resource-constrained devices and situations with low bandwidth, high latency, or unreliable networks. It operates on the publish/subscribe paradigm and is commonly used in the IoT and other scenarios where efficient, real-time communication is essential.
- Topics: In MQTT, messages are published according to topics, and subscribers express interest in certain topics by subscribing to them. Topics are hierarchical, using a forward slash (/) as a separator. For example, in our work, “iot/device1” or “iot/device2” are representative topic structures.
- Publishing: Publishers send messages to the broker, specifying a topic and payload. For example, in our work, a temperature and humidity sensor might publish a message to the topic “iot/device1”, with the payload being the actual temperature value.
- Subscribing: Subscribers express interest in specific topics by subscribing to them on the broker. Subscribers receive messages from topics they have subscribed to. For example, in our work, a subscriber receives messages by subscribing to the topic “iot/device2”, with the payload containing the actual temperature value.
- Broker Responsibilities: This is an intermediary server that manages the communication between publishers and subscribers. The broker receives messages from publishers and routes them to the appropriate subscribers based on topic subscriptions. It manages client connections, handles subscriptions, and delivers messages according to the specified Quality-of-Service (QoS) level.
- QoS Levels: MQTT supports different QoS [46] levels:
  ⇒   QoS 0: The message is delivered at most once, with no confirmation.
  ⇒   QoS 1: The message is delivered at least once, with confirmation.
  ⇒   QoS 2: The message is delivered exactly once by using a four-step handshake.
- Legitimate Traffic: Legitimate MQTT traffic involves authorized publishers and subscribers communicating with the broker. Publishers send messages to topics, and subscribers receive messages based on their topic subscriptions. Legitimate traffic is characterized by well-formed messages and adherence to the established topic structure.
- Malicious DDoS Traffic: Malicious entities may attempt to overload the MQTT broker by flooding it with connection requests or messages, leading to a Denial-of-Service situation.

#### 3.1.2. Smart Home IoT Device Sensors

**Figure 1:**
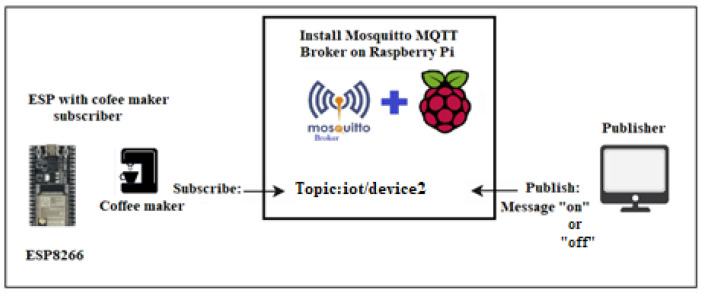
IoT device subscribes to a specific topic from the host publisher.

**Figure 2:**
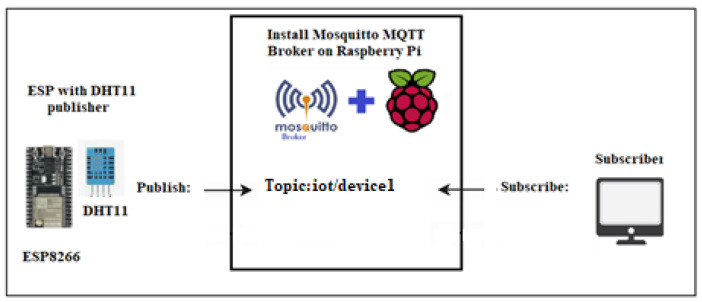
IoT device publishes a message to a specific topic for subscriber hosts.

Our smart home IoT device system includes a temperature and humidity sensor, along with a coffee maker sensor. They are configured as illustrated in Figure 1 and Figure 2. These two IoT devices are connected through ESP8266 and utilize the Mosquitto MQTT protocol. The Mosquitto MQTT server is installed on a Raspberry Pi desktop operating system of Raspberry Pi 4, enabling both publishing and remote control. MQTT facilitates messaging between host devices and the broker, as well as between the broker and IoT devices.

Temperature and humidity sensors are responsible for publishing temperature and humidity data, whereas coffee maker sensors are subscriber-controllable devices. The MQTT Mosquitto broker receives messages from publishers and directs them to the relevant subscribers based on the topic. Legitimate users or host devices can access temperature and humidity data and control the coffee maker device. Any suspicious or excessive network traffic is identified and categorized as potential DDoS attacks.

### 3.2. Types of Security Breaches in DDoS Attacks

**Figure 3:**
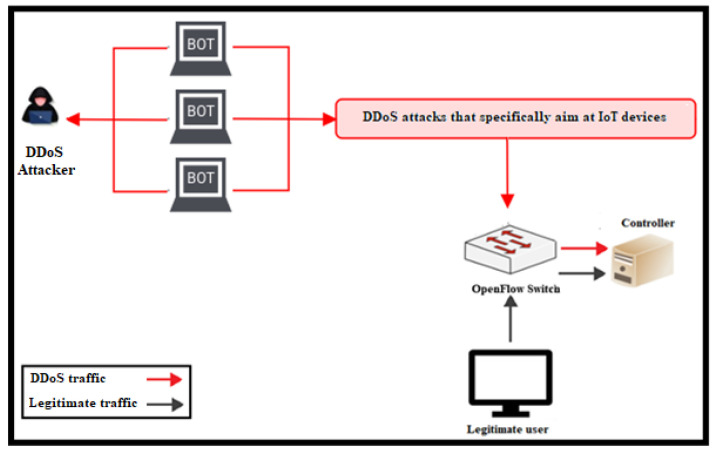
DDoS attacks in SDN.

As shown in Figure 3, DDoS attacks in SDNs are executed by generating numerous new flow entries that inundate the packet processing of OF switches and the controller, resulting in the unavailability of a network of IoT devices to users.

In 2023, DDoS attacks targeting IoT devices increased by 300%, as reported by references  [9,47,48]. This surge resulted in a global financial loss of 2.5 billion dollars during the first half of the year. Notably, 90% of the observed complex DDoS attacks in 2023 were orchestrated through botnets, exploiting the distributed nature of IoT devices such as routers, cameras, NAS boxes, and smart homes. These statistics underscore the escalating threat of DDoS attacks on IoT devices and emphasize the urgent need for enhanced security measures to safeguard these devices from potential cyber threats. Recent studies have identified CoAP Flood [49,50], MQTT broker DDoS attacks [51], HTTP flood, UDP flood, SYN flood, and ICMP flood [8,9,52] as the most perilous DDoS types targeting IoT devices, as illustrated in Table 2.

### 3.3. ML Algorithms

#### 3.3.1. k-Nearest Neighbors (kNN)

kNN retains the feature vectors of historical data points and, during training, memorizes the spatial relationships between instances in the feature space. When presented with a new instance, kNN identifies its k-nearest neighbors, and the majority class among these neighbors determines the classification of the instance.

#### 3.3.2. Naive Bayes (NB)

NB estimates the probability of each feature given the class labels (normal or DDoS attack) using historical data. When applied to predicted data, NB calculates the probability of the instance belonging to each class. The class with the highest probability is assigned as the final classification.

#### 3.3.3. Logistic Regression (LR)

LR assesses the likelihood of a DDoS attack based on historical features in the training data. It learns the coefficients for each feature in the logistic function. When applied to predicted data, LR calculates the probability of a DDoS attack. A predefined threshold is set, and instances with probabilities surpassing this threshold are classified as DDoS attacks.

#### 3.3.4. Random Forest (RF)

The RF algorithm discerns patterns in network features that distinguish normal traffic from DDoS attacks. It accomplishes this by constructing multiple DT, each trained on a subset of the data. Upon encountering a predicted data point, the RF ensemble collectively votes on its class. The mode of these votes determines whether the instance is classified as normal or indicative of a DDoS attack.

### 3.4. Integration of ML into SDN Architecture for Smart Homes

**Figure 4:**
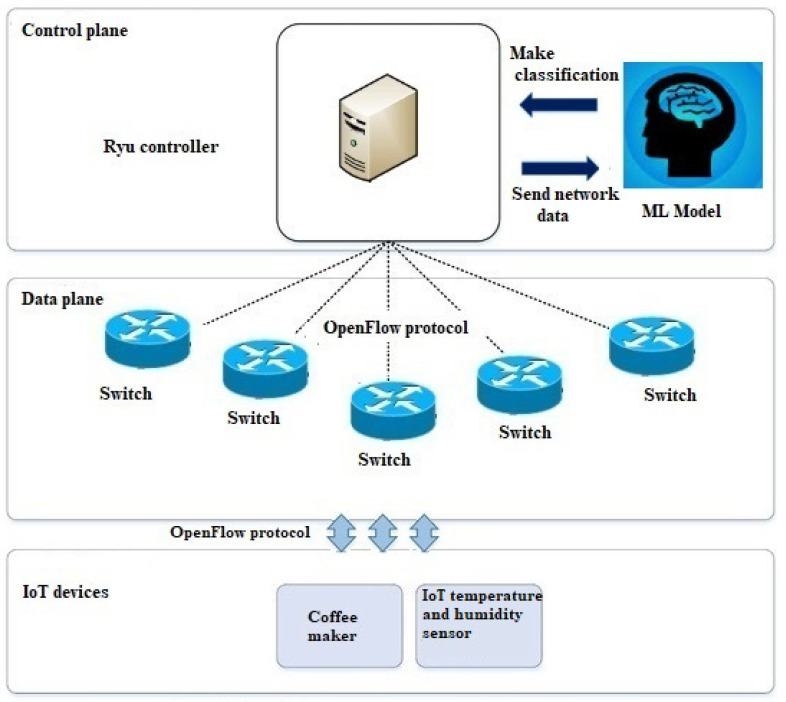
ML-based SDN Ryu controller framework for securing smart homes.

Figure 4 illustrates that the SDN Ryu controller serves as the central intelligence for network management. It communicates with the SDN switch using the OF protocol to control the flow of traffic to and from IoT devices and other hosts. The trained ML model is integrated into the Ryu SDN controller. This integration allows the ML model to analyze real-time network data received by the controller. As network traffic passes through the Ryu controller, the ML model classifies it in real time. The model assesses whether the current network behavior aligns with normal patterns or if there are indications of a potential DDoS attack. Based on ML classification, the Ryu controller can make dynamic decisions to mitigate the impact of DDoS attacks for IoT devices comprising a temperature and humidity sensor and a coffee maker sensor by reconfiguring network policies, redirecting traffic, or isolating affected devices.

## 4. Implementation

In this section, we will elucidate the tools employed and the SDN-ML-IoT methods that utilize ML techniques to ensure the security and stability of IoT devices within the SDN framework.

We will discuss the process step by step and evaluate the ML performance results to make an informed decision in selecting the SDN-ML-IoT framework.

### 4.1. Tools Used

As shown in Table 3, we employ a set of tools to facilitate various tasks in our project. Initially, we utilized a virtual machine (VM) with Ubuntu v20.04.1 to deploy Mininet and the Ryu controller for implementing network infrastructure. These tools aid in establishing a virtual network environment and efficiently managing network components. Additionally, we utilize Ryu controller tools to generate the dataset required for our research and analysis. We employ hping3 to simulate DDoS attacks, Mosquitto for publishing and subscribing to messages, and the Python programming language as the primary language for developing SDN applications and network control logic.

### 4.2. Collect Traffic Data

**Figure 5:**
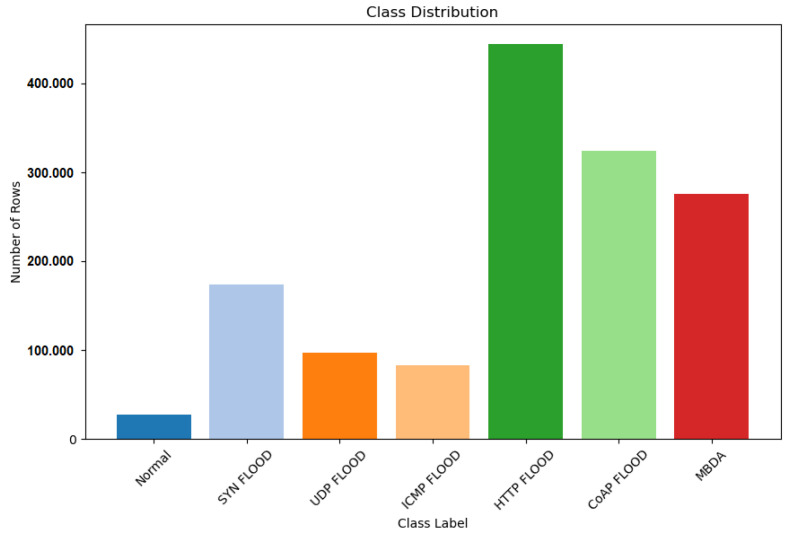
DDoS attack label details.

The Ryu controller is designed to efficiently collect network traffic data from host devices and IoT devices in a Mininet-based environment; the application monitors flow statistics in the SDN network. It collects flow statistics from OF-enabled network switches. The application periodically requests flow statistics from each switch and handles state changes in switches. When flow statistics replies are received, it extracts relevant features. The extracted data based on feature information are then written to a CSV file named “data.csv”. The application distinguishes between different IP protocols (ICMP, TCP, UDP). The monitoring interval is set to 10 s. It captures both normal and DDoS traffic. As shown in Figure 5, the collected dataset comprises six attack types and normal traffic, including normal, SYN flood, UDP flood, ICMP flood, HTTP flood, CoAP flood, and MQTT broker DDoS attacks. The CSV dataset contains a total of 1,426,858 records, representing various attacks and normal instances.

As indicated in Table 4, the dataset encompasses a total of 22 features, inclusive of the class label. The class label represents different types of attacks: normal, SYN flood, UDP flood, ICMP flood, HTTP/HTTPS flood, CoAP flood, and MQTT broker DDoS attacks, denoted by the values 0, 1, 2, 3, 4, 5, and 6, respectively.

### 4.3. Data Preprocessing

#### 4.3.1. Drop Duplicate Values

Any duplicate rows present in the dataset were removed to avoid redundant information.

#### 4.3.2. Label Encoding

We used the label encoding technique to transform categorical labels into numerical values. In this step, we applied label encoding to three specific features: flow_id, ip_src, and ip_dst. By employing label encoding, we converted these categorical data points into a numerical format suitable for ML algorithms. This transformation ensured that our dataset was well prepared for model training, preventing any challenges associated with handling non-numeric data. Ultimately, this process allows ML models to effectively learn from the data and make precise predictions.

### 4.4. Feature Selection

**Figure 6:**
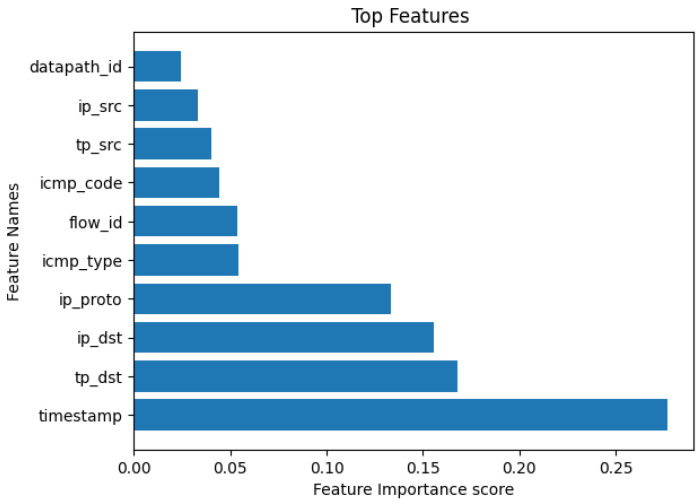
Top ten features selection using RFE module.

We utilized the Recursive Feature Elimination (RFE) [53] module for feature selection in our work. RFE is instrumental in the identification of the most pertinent features, thereby enhancing overall model performance. Through the elimination of less significant features, the model is able to concentrate on the most informative ones, subsequently mitigating noise within the data. In our specific work, when testing the model against real-time network traffic. As shown in Figure 6, we selected the top 10 important features, employing fewer than 10 features often results in an elevated false alarm rate. Conversely, selecting more than 10 features has the potential to induce overfitting, where the model excels in training data but struggles to generalize to novel, unseen data. RFE plays a crucial role in averting overfitting by meticulously choosing a subset of features that maximally contribute to predictive performance.

### 4.5. One-versus-Rest (OvR) Strategy Setup

We used the OvR strategy in multiclass classification tasks to simplify the problem and leverage binary classification algorithms. The training of binary classifiers makes it computationally efficient, especially for large datasets. This parallelization can lead to faster training times. Below is an explanation of how OvR works for our dataset label class:

For each class i∈C, train a binary classifier $h_{i}$ with the following labels:

$$\begin{array}{cc} \mathrm{Positive}\;\mathrm{class}: & \;+1\;:\;\mathrm{Class}\;Normal(0)\\ \mathrm{Negative}\;\mathrm{class}: & \;-1\;:\;\mathrm{All}\;\mathrm{other}\;\mathrm{six}\;\mathrm{DDoS}\;\mathrm{classes}\;(\mathrm{from}\;1\;\mathrm{to}\;6) \end{array}$$

For each binary classifier $h_{i}$, the training involves learning a model $f_{i}$ to distinguish between instances of class *i* and instances not belonging to class *i*. The training process minimizes a binary classification loss function for each classifier.

Mathematically, the prediction for a binary classifier $h_{i}$ is given by:

(1) $$h_{i}\left(x\right)=\left\{\begin{array}{cc} +1 & \mathrm{if}\;f_{i}\left(x\right)\geq 0\\ -1 & \mathrm{if}\;f_{i}\left(x\right)<0 \end{array}\right.$$

To predict the class for a new instance x, evaluate each binary classifier $h_{i}$ and choose the class associated with the classifier that produces the highest score. The predicted class is given by:

(2) $$\hat{y}=\arg\max_{i\in C}f_{i}\left(x\right)$$

### 4.6. Data Splitting

The transformed feature data $X_{F}$ and the target variable y were split into training and testing sets. A standard practice involves allocating 75% of the data for training and reserving 25% for testing purposes.

### 4.7. Balancing Data Classes

Following the data split, we employed undersampling techniques [54] to achieve a balanced class distribution within the training set. Undersampling entails the random removal of instances from the majority class, aligning it more closely with the minority class. It is essential to emphasize that this step is exclusively applied to the training data to prevent any potential data leakage. We opted for undersampling techniques over SMOTE [55] due to concerns of bias and false alarms. Undersampling techniques provide more accurate results.

### 4.8. Model Training

After collecting the dataset, as explained in Section 4.2, we divided it into training and testing sets. The OvR strategy was applied to transform the multiclass classification problem into binary classification. To achieve a balanced binary class distribution, undersampling was employed, and only the top 10 important features were selected. The ML model, incorporating kNN, NB, LR, and RF algorithms, was then trained using the selected features from the training set. Following the training phase, the model underwent testing on the test dataset to assess its ability to make accurate predictions on new, unseen flow data. Based on grid search cross-validation (GridSearchCV) [56] to find the optimal set of hyperparameters, the parameters employed for each ML algorithm are outlined below in Table 5.

Our SDN-ML-IoT-based system incorporates the OvR strategy. In our specific scenario, the objective is to predict among seven classes, while numerous classification algorithms are inherently designed for binary classification, distinguishing only between normal and DDoS attacks and capable of handling two classes at a time. The OvR strategy proves to be a practical solution in addressing our multi-class classification problem.

Cross-validation using k-fold: Cross-validation was implemented using k-fold validation [57] with k = 10 to assess the model’s robustness and generalization across different subsets of the training data. This ensures a more comprehensive evaluation of the model’s performance and helps identify potential overfitting or underfitting issues.

### 4.9. Model Evaluation

Finally, we evaluated the model’s performance using various metrics on the testing dataset. The key evaluation metrics included accuracy, precision, AUC-ROC, training time, and prediction time. These metrics provide a holistic view of the model’s effectiveness in making accurate predictions and its computational efficiency. The evaluation metrics are listed and defined below:

(3) $$ConfusionMatrix=\left[\begin{array}{cc} TP & FN\\ FP & TN \end{array}\right]$$

(4) $$Accuracy=\frac{TP+TN}{TP+FN+FP+TN}\;$$

(5) $$Sensitivity=\frac{TP}{TP+FN}$$

(6) $$Specificity=\frac{TN}{TN+FP}$$

where:

*N*: The number of target classes in the dataset.

True Positives (*TP*s): The instances where the model correctly identified a rule and detected an attack.

False Positives (*FP*s): The instances where the model incorrectly identified a rule and classified an instance as an attack when it was not present.

True Negatives (*TN*s): The instances where the model correctly identified that no rule matched and correctly classified an instance as not being an attack.

False Negatives (*FN*s): The instances where the model incorrectly identified that no rule matched and failed to detect an attack.

The Area Under the Receiver Operating Characteristic curve (AUC-ROC): A performance metric for binary classification models. It evaluates the trade-off between sensitivity (true positive rate) and specificity (true negative rate). The ROC curve plots the true positive rate against the false positive rate at various threshold settings. AUC-ROC quantifies the classifier’s ability to distinguish between classes, with a higher AUC indicating better overall performance, considering both sensitivity and specificity.

### 4.10. Simulation Results

Table 6 displays the training results for RF, LR, kNN, and NB. The accuracy metric indicates strong performance for both RF and kNN. RF attains a high accuracy of 0.9999 and an AUC-ROC of 0.9999; however, it requires longer fit and testing times compared to the other algorithms. kNN demonstrates commendable accuracy at 0.9998 and a perfect AUC-ROC of 0.9999, with shorter times spent on training and testing compared to RF. In contrast, NB and LR exhibit suboptimal accuracy, suggesting that they may not be the most suitable models for this specific task.

LR and NB were omitted from our model selection in favor of RF and kNN, which produced superior results. This decision led us to proceed with RF and kNN for the subsequent simulation test in a real SDN Testbed.

We conducted tests on various network topologies—single, linear, tree, ring, and mesh structures—with different sizes—small (4 hosts), medium (16 hosts), and large (64 hosts). To initiate the deployment process for our proposed framework SDN-ML-IoT, we integrated the Ryu controller with our ML models, which are based on kNN-OvR or RF-OvR. The deployment also involved using Mininet to establish network configurations. These configurations are detailed in Table 7.

#### 4.10.1. Evaluating SDN Performance Based on Detection Time

We conducted comparative studies on multiple network topologies and sizes to assess the SDN performance within RF and KNN, specifically focusing on detection time. Table 8 illustrates that kNN has a lower detection time compared to RF for all network topology types and sizes. The detection time results for RF and kNN exhibited nearly identical detection rates for DDoS attacks. Based on a comparison of the two algorithms, we conclude that across small, medium, and large configurations, the detection times are almost similar. However, the mesh topology displayed a longer detection time of more than 2 s due to its inherent complexity.

#### 4.10.2. Evaluating SDN Performance Based on CPU Utilization and Memory Usage

Based on the last comparison, we conclude that single linear, tree, and ring topologies are similar, unlike mesh topology, owing to their complexity, redundancy, and highly interconnected switches. Therefore, we continue evaluation based on the two SDN topologies: linear topology and mesh topology. Table 9, Table 10 and Table 11 present evaluations of CPU usage and memory consumption for linear and mesh topology scenarios. Table 9 focuses on configurations with four hosts, four switches, and one controller. In Table 10, the evaluation extends to setups with 16 hosts, 16 switches, and 1 controller. Lastly, Table 11 examines CPU usage and memory consumption for larger networks featuring 64 hosts, 64 switches, and 1 controller. The results indicate that kNN exhibits higher memory consumption than RF, especially for large topologies and complex networks. The RF algorithms demonstrate a more significant reduction in CPU and memory usage after mitigating DDoS attacks compared to kNN.

#### 4.10.3. Model Selection

The model selection for integration on live SDN monitoring traffic is directed toward RF due to its superior accuracy, acceptable fit and prediction times, and flexibility with multiple SDN topologies, along with lower memory consumption and CPU usage. The integration using the RF model can be scaled and adapted to different network sizes and types.

### 4.11. Model Integration

After demonstrating the scalability, adaptability, and reliability of our SDN-ML-IoT framework across various topology types such as single, linear, tree, ring, and mesh, as well as different sizes, including small, medium, and large, as detailed in Section 3.4, we integrated our model using the Ryu controller along with the switching application. We employed Mininet to establish an SDN with diverse topology types, including single, linear, tree, ring, and mesh, and varying sizes from small to large. During live traffic, the switch forwards packet information to the ML classifier integrated into the Ryu controller. Our SDN-ML-IoT framework, which is based on RF-OvR, determines whether the traffic is malicious. For legitimate traffic, the controller examines the packet’s destination and makes a decision on the output port. Subsequently, it adds a new rule to the forwarding layer to permit the traffic. In the case of malicious traffic, the controller instructs the forwarding layer to block packets by sending a rule that creates a flow entry to drop the packet.

## 5. Framework Deployment on a Real Testbed

In this section, we emphasize the steps involved in integrating SDN-ML-IoT into a real SDN topology. We discuss the deployment process on an actual live network and subsequently assess their effectiveness across various topology types.

### 5.1. Detection and Mitigation of DDoS Attacks

**Figure 7:**
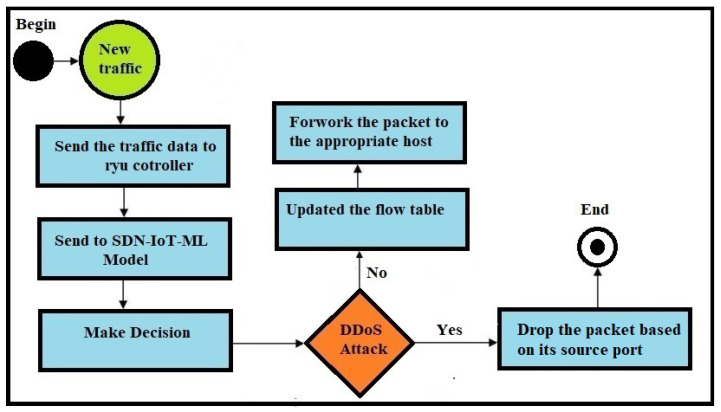
Activity diagram of SDN-ML-IoT method to monitor traffic.

Figure 7 depicts the operational diagram of the SDN-ML-IoT method within an SDN topology. This method serves as a controller, enabling real-time decision making to differentiate between legitimate traffic and the presence of DDoS attacks. If the traffic is deemed legitimate, it forwards the packets to the appropriate host device or IoT device; otherwise, it blocks the packets.

Algorithm 1 detects DDoS attacks based on a list of predictions (flow_pred) related to network traffic flows. It counts legitimate and DDoS traffic within the predictions and computes a label class for non-zero values. If a significant portion of the traffic is legitimate (more than 90%), it classifies the traffic as legitimate (assigns prediction as 0). If not, it uses a label class to classify the traffic as a DDoS attack (assigns predict as 1) and returns this classification as predict.
**Algorithm** **1** DDoS attacks detection  **Input:** flow_pred  **Output:** predict  legitimate_traffic←0  **Begin:**  **for** *i* **in** flow_pred:    **if** i == 0:      legitimate_traffic←legitimate_traffic+1    **end if**  **end for**  **if** (legitimate_traffic/len(flow_pred))>0.9:    predict←0  **end if**  **else:**    predict←1  **end else**  **return** predict  **end**

Algorithm 2 identifies the source port and network device of the incoming packet. Subsequently, it takes the action of dropping any packets arriving on the specified source port of the given network device for a duration of 100 s.
**Algorithm** **2** Incoming packet identification  **Input:** packet_received  **Output:** block DDoS attacks  **Begin:**  source_port = packet_received.source_port  datapath_id = packet_received.datapath_id  **if** is_ddos_attack(packet_received):
      Send a flow modification message to the switch to drop packets from the identified source port with a timeout of 100 s
   **end if**  **end**

### 5.2. Legitimate Traffic

**Figure 8:**
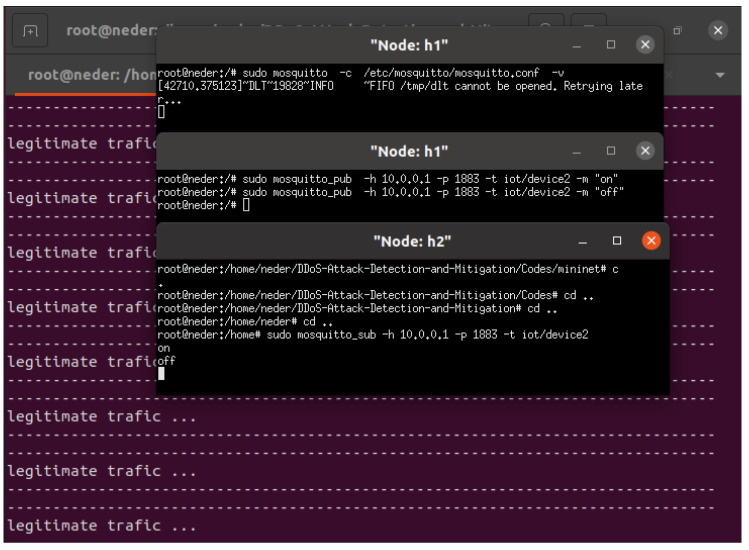
Subscribe to legitimate traffic for smart coffee IoT device.

**Figure 9:**
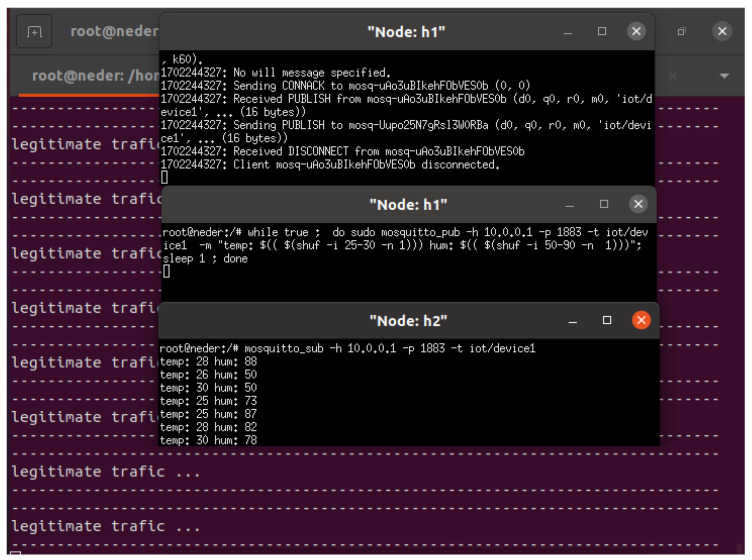
Temperature and humidity IoT device publishes legitimate traffic.

- In the case of the smart coffee maker, legitimate traffic would involve sending an “on” or “off” payload message from an h1 to the IoT device smart coffee maker, which is h2, to control its power state. This communication allows h1 users to remotely turn the coffee maker on or off based on their preferences. Figure 8 shows the scenario of legitimate traffic in which h2 (smart coffee) subscribes to h1, enabling remote control of the coffee maker based on its needs. Our SDN-ML-IoT framework accurately predicts legitimate traffic originating from h1 to IoT device subscribers.
- In the case of the temperature and humidity IoT sensor, legitimate traffic involves the IoT device acting as h1, transmitting temperature and humidity data to h2. Then, h1, functioning as an IoT sensor, publishes temperature and humidity data, while h2 subscribes to h1 to collect the data from h1. Figure 9 depicts the scenario of legitimate traffic in which h1 (the temperature and humidity sensor) publishes temperature and humidity data to subscriber h2. Our SDN-ML-IoT framework accurately predicts legitimate traffic originating from IoT publishers.

### 5.3. DDoS Attacks

**Figure 10:**
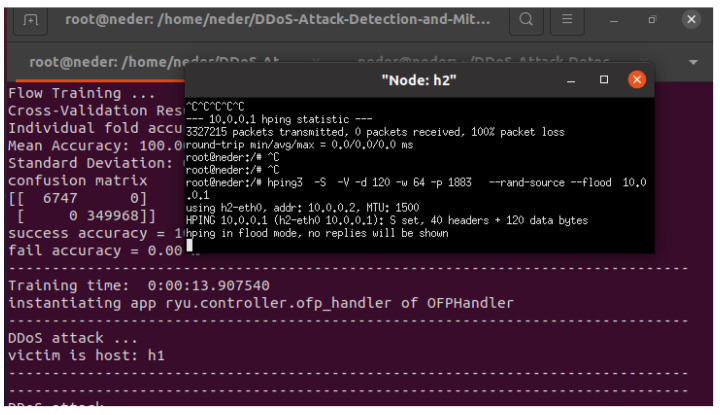
Detecting DDoS attacks targeting IoT devices.

The hping3 tool is widely recognized as a frequently used utility in DDoS attacks, as indicated in Table 3. As depicted in Figure 10, h2 utilizes hping3 to generate a substantial volume of packets directed toward the target machine, h1, which is an IoT device targeting the MQTT port. Our SDN-ML-IoT framework detects DDoS attacks in less than 3 s.

### 5.4. Blocking DDoS Attacks

**Figure 11:**
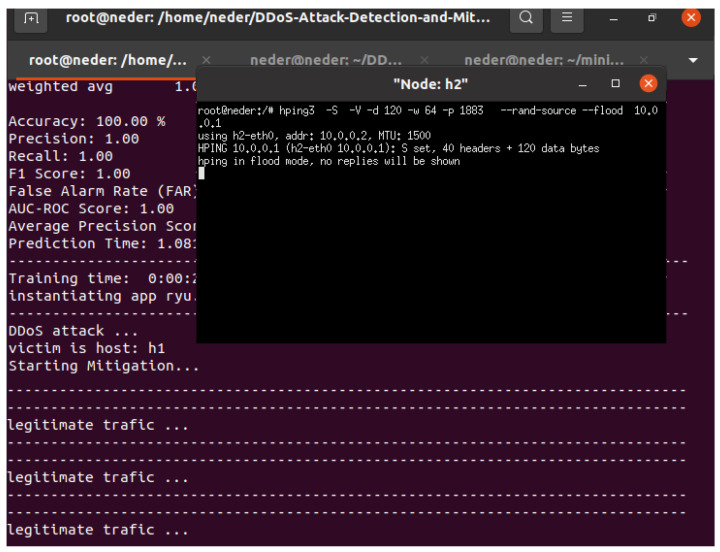
Mitigating DDoS attacks targeting IoT devices.

As illustrated in Figure 11, the processes for mitigating DDoS attacks using packet switching and their corresponding source port function effectively, successfully blocking DDoS attacks in real time.

## 6. Results and Discussion

This section presents a comparative study of three related works closely aligned with my research and our SDN-ML-IoT method. As shown in Table 12 below, Zhenpeng Liu, in [30], employed an improved binary grey wolf optimization algorithm and RF. Their model achieved an accuracy of 99.13%. When compared to similar studies, the presented work demonstrated an improvement in accuracy by 0.0033. Hani Elubeyd, in their paper [37], proposed a hybrid deep learning model that combines three algorithms: a 1D CNN, a GRU, and a DNN. They achieved an accuracy of 99.81%, improving upon other related works by 0.50%. Walid I. Khedr, in the paper [38], utilized the FMDADM framework based on the RF algorithm, achieving an accuracy of 99.79%. This outperforms previous related works by 0.08%.

Our proposed method, SDN-ML-IoT, employs the RF algorithm on a synthetic dataset. It is essential to note that different studies use diverse datasets, models, and evaluation metrics. Consequently, making direct comparisons with the results of other studies can be challenging. Nonetheless, our proposed method exhibits outstanding accuracy, achieving 99.99%. This performance surpasses that of related studies. It adeptly detects DDoS attacks on SDNs and effectively mitigates these attacks.

## 7. Conclusions and Future Works

This paper introduces an enhanced IDPS framework, utilizing the RF algorithm within the SDN framework, named SDN-ML-IoT, aimed at fortifying the security of IoT devices in smart homes against DDoS attacks. The model selection process involved the collection of a synthetic dataset based on the monitoring capabilities of the Ryu controller. This dataset encompasses normal traffic and six distinct types of DDoS attacks, tailored to the specific requirements of IoT devices in smart homes. The dataset was then utilized to train and evaluate four ML algorithms specialized in IDPS: NB, LR, KNN, and RF. To address the multiclass classification challenge, we employed an OvR strategy to transform it into a binary classification problem, optimizing the detection problem for binary classification. This strategy facilitated the handling of imbalanced data, reduced computational complexity, and improved training and prediction times. Additionally, we utilized the REF method to streamline feature selection, reducing training time and enhancing accuracy. The method also incorporated a method and fold cross-validation approach to mitigate overfitting. The simulation results showed that the selection of RF as the SDN-ML-IoT framework was favorable for real-time deployment within SDN networks for smart homes, achieving an accuracy of 99.99% and a training time of 20 s. The model demonstrated adaptability and effectiveness across different network topologies and sizes, providing predictive detection times between 1 and 3 s, depending on network complexity. The SDN-ML-IoT not only identifies DDoS attacks but also mitigates them by blocking the DDoS packets based on their source ports.

In future work, we plan to implement our SDN-IoT-ML framework in real-world deployments to evaluate its results. We aim to enhance our model by incorporating multiclass classification to directly mitigate attacks based on their class, leveraging the ip_proto field. Additionally, we will focus on exploring other attack types targeting IoT devices, emphasizing threats such as man-in-the-middle attacks, Botnets, Zero-Day Exploits, and more. Employing multiple Ryu controllers will facilitate the rapid sharing of threat information, enabling controllers to respond more quickly to emerging security threats. This investigation aims to enhance the understanding of security vulnerabilities in IoT systems and develop robust countermeasures against these prevalent threats.

## Figures and Tables

**Table 1:** Summary of the related works.

Research	Advantages	Limitations
[16]	Lower false positive rates and faster response times.	Mitigation of DDoS attacks after detecting. Improved accuracy.
[19]	Effective differentiation of malicious attacks using the SATIDS method and precise categorization of attacks.	Accuracy improvements. Mitigation of DDoS attacks after detection.
[24]	Gini-impurity-based approach that achieves high accuracy. Deployment on live SDN traffic. Mitigation.	Reduces the number of features.
[30]	Binary grey wolf optimization algorithm for feature engineering. High accuracy.	Reduces the number of features. The performance of the proposed method was tested on diverse network sizes and types.
[35]	Deployment on SDN Network. Mitigation.	Overfitting in accuracy results.
[36]	Reduces features. Mitigation.	Gathers additional data from alternative protocols. The performance of the proposed method was tested on diverse network sizes and types.
[37]	High accuracy with low False Alarm Rate (FAR) and minimal detection time. Mitigation.	The proposed method was tested on live SDN traffic. The performance of the proposed method was tested on diverse network sizes and types.
[38]	A combination of three different types of neural network layers was utilized, yielding high accuracy.	Evaluation of a specific dataset. Mitigation of DDoS attacks after detection. The proposed method was teseted on live SDN traffic.

**Table 2:** The most dangerous DDoS attacks targeting IoT devices.

DDoS Attack Names	Type	Target	Vulnerability
SYN flood	Network-based DDoS attack.	TCP/IP handshake process.	IoT devices, due to limited computational and networking capabilities.
UDP flood	Floods the target’s network with UDP packets.	Overwhelms network resources.	IoT devices can be used as amplifiers to multiply traffic volume.
ICMP flood	Floods the target with ICMP packets.	Consumes resources, potentially causing network congestion.	IoT devices susceptible to resource exhaustion.
HTTP flood	Floods the target’s web server with HTTP/HTTPS requests.	Exhausts server resources, making it difficult for legitimate users.	Impacts IoT devices with web server functionality.
MQTT broker DDoS attacks	Targets MQTT brokers used in IoT environments.	Saturates connections to and from the MQTT broker.	Overwhelms MQTT brokers, impacting IoT functionality.
CoAP flood	Targets IoT devices using CoAP, a lightweight protocol.	Overwhelms devices with a high volume of CoAP requests.	Disrupts the operation of CoAP-enabled IoT devices.

**Table 3:** Tools and their descriptions used for implementing SDN.

Tool Name	Description
Mininet	A network emulator that creates a virtual network environment consisting of hosts, switches, controllers, and links.
Ryu controller	A software framework with well-defined APIs that simplify the development of network management and control applications. It is fully written in Python.
Oracle VM	Cross-platform virtualization software, Virtual Box, enabling users to run multiple operating systems, such as Microsoft Windows, Mac OS X, and Linux, on their existing computers. In our work, it enables us to run Ubuntu.
Ubuntu v20.04.1	The operating system used on the VM. Ubuntu is a popular choice as the underlying operating system on which other SDN-related software, such as the Ryu controller and Mininet, can be easily installed and utilized.
Python3	A programming language utilized for creating the Mininet topology and developing the Ryu controller.
Mosquitto	An open-source message broker that implements the MQTT. It is used as a central server that facilitates communication between MQTT clients, allowing them to publish and subscribe to topics, exchange messages, and coordinate data transfer in a scalable and efficient manner.
hping3	A network tool capable of generating flooding attacks.

**Table 4:** The features of collected datasets.

ID	Feature Name	ID	Feature Name
1	Timestamp	12	flow_duration_nsec
2	datapath_id	13	idle_timeout
3	flow_id	14	hard_timeout
4	ip_src	15	Flags
5	tp_src	16	packet_count
6	ip_dst	17	byte_count
7	tp_dst	18	packet_count_per_second
8	ip_proto	19	packet_count_per_nsecond
9	icmp_code	20	byte_count_per_second
10	icmp_type	21	byte_count_per_nsecond
11	flow_duration_sec	22	Label

**Table 5:** Parameters employed in ML algorithms for our SDN-ML-IoT model.

ML Algorithm	Parameters
RF	n_estimators = 100, criterion = “entropy” and random_state = 0.
LR	C = 1.0, penalty = “l2”, solver = “lbfgs” and max_iter = 100.
kNN	n_neighbors = 5.
NB	Default Parameters.

**Table 6:** Evaluation metrics results.

ML Methods	Training Time (Sec)	Prediction Time (Sec)	Confusion Matrix	Accuracy	AUC-ROC
RF	25.95	1.1656	$\left[\begin{array}{cc} 6747 & 0\\ 1 & 349967 \end{array}\right]$	0.9999	0.9999
LR	09.47	0.0063	$\left[\begin{array}{cc} 6747 & 0\\ 349968 & 0 \end{array}\right]$	0.5000	0.5000
kNN	13.96	1.0050	$\left[\begin{array}{cc} 6747 & 0\\ 155 & 349813 \end{array}\right]$	0.9998	0.9999
NB	05.73	1.074	$\left[\begin{array}{cc} 6747 & 0\\ 138670 & 211298 \end{array}\right]$	0.6113	0.9658

**Table 7:** Configuration details for SDN encompassing multiple topology types and sizes.

Network Topology Type	Number of Hosts	Number of Switches	Number of Controllers
Single network size	4	1	1
	16	1	
	64	1	
Linear network size	4	4	1
	16	16	
	64	64	
Tree network size	4	3	1
	16	15	
	64	63	
Ring network size	4	4	1
	16	16	
	64	64	
Mesh network size	4	4	1
	16	16	
	64	64	

**Table 8:** Evaluation of the detection time for multiple topology types and sizes using RF and KNN.

Network Topology Type	Network Size	Detection Time Using RF (ms)	Detection Time Using kNN (ms)
Single network size	4	1222	1113
	16	1387	1282
	64	1407	1314
Linear network size	4	1184	1129
	16	1222	1131
	64	1281	1269
Tree network size	4	1333	1086
	16	1599	1178
	64	1701	1234
Ring network size	4	1098	1012
	16	1152	1288
	64	1198	1311
Mesh network size	4	1713	1612
	16	1921	1888
	64	2081	2011

**Table 9:** Evaluation of CPU usage and memory consumption for linear and mesh topology types with a small size using RF and kNN.

ML Methods	CPU Usage Under DDoS Attacks	Memory Consumption Under DDoS Attacks	CPU Usage after Mitigation of DDoS Attacks	Memory Consumption after Mitigation of DDoS Attacks
RF linear topology	55.67%	37.58%	8.71%	32.68%
kNN linear topology	65.98%	41.51%	10.24%	39.47%
RF mesh topology	76.58%	33.99%	18.79%	22.80%
kNN mesh topology	68.77%	41.46%	32.69%	38.73%

**Table 10:** Evaluation of CPU usage and memory consumption for linear and mesh topology types with a medium size using RF and kNN.

ML Methods	CPU Usage Under DDoS Attacks	Memory Consumption Under DDoS Attacks	CPU Usage after Mitigation of DDoS Attacks	Memory Consumption after Mitigation of DDoS Attacks
RF linear topology	78.70%	34.22%	20.10%	33.52%
kNN linear topology	84.33%	41.59%	40.06%	40.87%
RF mesh topology	88.36%	28.42%	16.00%	28.78%
kNN mesh topology	88.92%	37.21%	65.98%	35.37%

**Table 11:** Evaluation of CPU usage and memory consumption for linear and mesh topology types with a large size using RF and kNN.

ML Methods	CPU Usage Under DDoS Attacks	Memory Consumption Under DDoS Attacks	CPU Usage after Mitigation of DDoS Attacks	Memory Consumption after Mitigation of DDoS Attacks
RF linear topology	96.49%	35.11%	24.47%	40.46%
kNN linear topology	92.16%	42.34%	35.43%	40.52%
RF mesh topology	99.49%	36.97%	23.79%	27.80%
kNN mesh topology	99.36%	49.96%	83.35%	39.50%

**Table 12:** Comparison of our method with other studies.

Research	Method Used	Accuracy	Mitigation	Environment Used	Topology Network Adaptable
[30]	RF method.	99.9%	Using in_port.	Mininet and Ryu controller.	Tree Topology.
[37]	Hybrid deep learning approach.	99.81%	X	Mininet and Ryu controller.	Not specified.
[38]	RF method.	99.79%	Using src_port.	Mininet and POX controller.	Not evaluated.
SDN-ML-IoT	RF method.	99.99%	Using src_port with 100 sec timeout.	Mininet and Ryu controller.	Single, linear, tree, ring, and mesh.

## Data Availability

Data are contained within the article.
